# Dintor: functional annotation of genomic and proteomic data

**DOI:** 10.1186/s12864-015-2279-5

**Published:** 2015-12-21

**Authors:** Christian X. Weichenberger, Hagen Blankenburg, Antonia Palermo, Yuri D’Elia, Eva König, Erik Bernstein, Francisco S. Domingues

**Affiliations:** Center for Biomedicine, European Academy of Bolzano/Bozen (EURAC), (Affiliated to the University of Lübeck, Lübeck, Germany), Viale Druso 1, 39100 Bolzano, Italy; Deutsches Krebsforschungszentrum (DKFZ), Im Neuenheimer Feld 280, 69120 Heidelberg, Germany

**Keywords:** Reproducible research, Data integration, Data versioning, Gene prioritization, Gene set enrichment, Protein functional similarity, Genomics and proteomics pipeline, Galaxy web server

## Abstract

**Background:**

During the last decade, a great number of extremely valuable large-scale genomics and proteomics datasets have become available to the research community. In addition, dropping costs for conducting high-throughput sequencing experiments and the option to outsource them considerably contribute to an increasing number of researchers becoming active in this field. Even though various computational approaches have been developed to analyze these data, it is still a laborious task involving prudent integration of many heterogeneous and frequently updated data sources, creating a barrier for interested scientists to accomplish their own analysis.

**Results:**

We have implemented Dintor, a data integration framework that provides a set of over 30 tools to assist researchers in the exploration of genomics and proteomics datasets. Each of the tools solves a particular task and several tools can be combined into data processing pipelines. Dintor covers a wide range of frequently required functionalities, from gene identifier conversions and orthology mappings to functional annotation of proteins and genetic variants up to candidate gene prioritization and Gene Ontology-based gene set enrichment analysis. Since the tools operate on constantly changing datasets, we provide a mechanism to unambiguously link tools with different versions of archived datasets, which guarantees reproducible results for future tool invocations. We demonstrate a selection of Dintor’s capabilities by analyzing datasets from four representative publications. The open source software can be downloaded and installed on a local Unix machine. For reasons of data privacy it can be configured to retrieve local data only. In addition, the Dintor tools are available on our public Galaxy web service at http://dintor.eurac.edu.

**Conclusions:**

Dintor is a computational annotation framework for the analysis of genomic and proteomic datasets, providing a rich set of tools that cover the most frequently encountered tasks. A major advantage is its capability to consistently handle multiple versions of tool-associated datasets, supporting the researcher in delivering reproducible results.

**Electronic supplementary material:**

The online version of this article (doi:10.1186/s12864-015-2279-5) contains supplementary material, which is available to authorized users.

## Background

Analysis of high-throughput genomic and proteomic datasets requires familiarity with the use of specialized tools and web servers, and heterogeneous, complex data from various databases. This is often a barrier for interested researchers [1]. Furthermore, external databases and web servers are undergoing constant update cycles, leaving the analyst with the burden to archive and version data for reproducibility of results [2, 3]. Numerous solutions have been developed to assist researchers in data exploration, mostly published as web services, with notable efforts to generate workflow management systems that are able to integrate command line tools or other web server’s data [4–9], as part of a programming environment [10], or as stand-alone programs [11–13]. In many of these efforts emphasis is put on solving a specific problem, such as gene set analysis [14, 15] or disease gene prioritization [16], with less importance put on documentation and management of underlying data. Web-based solutions in particular usually do not provide any means for archiving the source data that were used in the computations, with the result that analyses cannot be reproduced once the web server is updated. Conversely, some approaches are dedicated to prudent data handling by employing data warehousing methodologies (briefly reviewed in [17]), but by definition offer little means for data analysis.

**Fig. 1:**
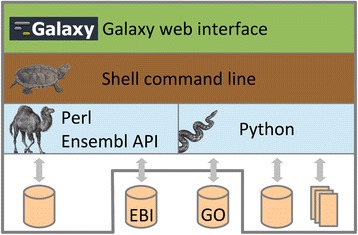
Hierarchical setup of the Dintor framework. On the top level, a Galaxy web server provides access via a graphical user interface to all available tools. The web interface is built on a collection of Unix shell command line tools, which come with detailed help pages. These tools can further be separated into two large subgroups: one is dedicated to querying the Ensembl database and employs Perl as a programming language. The other subgroup contains the remaining modules, which are implemented in Python. All modules are characterized by accessing either external or internal relational databases such as Ensembl or the Gene Ontology database, or operate on locally stored text files provided with the distribution. For privacy or performance reasons it is possible to configure Dintor such that it accesses only local data. The associations of these animal drawings with the respective programming languages are the protected trademarks of O’Reilly Media, Inc. Used with permission

Consistent use of software alongside well-defined versions of underlying data is highly appreciated in long-standing collaborations or when addressing questions raised during the review process of a submitted manuscript. Therefore, our goal was to develop a tool suite that facilitates exploration and analysis of large-scale genomic and proteomic datasets and that provides means to easily identify and exchange the underlying datasets used by the analysis tools. The resulting Dintor framework consists of more than 30 tools, based on well accepted, published, and popular methods that provide rich functionality for handling single nucleotide polymorphism (SNP) data; gene, protein, and transcript identifier conversions; functional annotation of SNPs and genes; gene prioritization and gene set enrichment analysis; as well as biomedical annotation. The framework distinguishes itself from similar approaches by employing versioned data files as tool data sources, which assures reproducible results for tool invocations at any time in the future. This is especially important when working on long-term projects, where results from data analysis are used as feedback to experimental designs, which then produce the next generation of data to analyze. The tools can be executed on our Galaxy [5] web server, http://dintor.eurac.edu, and for reasons of performance, privacy or data security they can be installed and run locally on Unix-like environments, with the additional option to set up a local mirror of the Galaxy sever (see Fig. 1). The Dintor framework is an open, extensible, and easy to use solution for reproducible functional analysis of genomics and proteomics data.

## Implementation

The tool suite has been designed as an open and modular framework with equal importance given to both functionality and dataset versioning. The tools are intended to be invoked in pipelines and therefore were planned as independent and reusable units that fulfill a special purpose in order to achieve a certain, more complex goal. Tools are given a high degree of flexibility for accessing data by allowing users to define specific versions of their underlying data resources. This is implemented with a global, version-aware, and extensible configuration directory. Each Dintor release comes with its own configuration directory that explicitly links data files and databases with tools. Versioning is guaranteed by storing data files and databases with time stamps. Each time a tool requests access to data, it is pointed to the appropriate data source through the configuration directory. We have chosen Ensembl as the principal source of data for our framework, since their Biomart data warehouse [18, 19] provides a comprehensive and regularly updated resource for genomic data.

The modules have been implemented in the programming languages Python and Perl, the latter is required by the Ensembl application programming interface (Fig. 1). Dintor tools process input as tabular text files and generally append resulting columns to each row without altering the content of the input rows. Thus, information can be passed on seamlessly between tools in a pipeline-like manner. Each tool is accessible via a consistent command line interface, which is wrapped by a Galaxy server. Therefore, the framework addresses both graphical interface-oriented users through the web server and bioinformaticians through the command line tools. In both cases, pipelines are built with either the graphical interface of the Galaxy tools or by utilizing a Unix shell script harnessing the command line tools.

One of the added values of the framework is that each tool is tied to a set of predefined and well-documented data files. Therefore, the origin and date of the different data sources are always defined when running a tool. We have stored updates for the data sources of the different tools since their implementation up to the last GRCh37 release of the Ensembl genome browser (Ensembl 75). Our earliest datasets range back to Ensembl 65 so that users can reproduce previous results. For example, Human Genome Organisation Gene Nomenclature Committee (HGNC) gene symbols, which frequently change names, can easily be queried with their identifiers used at any point of time during the last three years. The Dintor release cycle is following the update cycle of the Ensembl database, providing a self-contained set of tools and data. To gain maximum flexibility, any tool can ultimately be furnished with user-defined data files.

We assure high standards of code stability and quality by running more than 800 unit tests on a nightly basis. Each of the 30 tools comes with a concise description on the command line level and with extensive documentation in the Galaxy framework, including example invocations and a tutorial explaining selected tools and workflows. An overview of all Dintor tools is given in Additional file 1.

The data integration framework is split logically into the following groups: (1) basic functionality, for dealing with gene location and gene identifier conversions; (2) annotation retrieval, where information relevant to given variants and genes is retrieved from established function-related databases; and (3) computational methods, which are used to investigate relationships between genes, gene products, and within gene sets.

### Basic functionality

Variation data represent the core of many genomic analyses. Therefore, we offer a set of tools that provide an interface to dbSNP [20] and SNP-related data. Information such as the location on the chromosome, alleles, Ensembl validation state, and conservation scores are readily at the hands of the researcher. For pairs of SNPs it is possible to compute linkage disequilibrium (LD) expressed either as *D’* or *r*^2^ scores [21]. If the variation locates within a gene then variation consequences and deleteriousness measures [22–24] are accessible on a transcript basis. Locations may also be queried for overlaps with Ensembl regulatory regions [25].

Genes are frequently reported in different identifier systems, which hinders establishing connections between heterogeneous datasets. We supply conversion tools between the most widely used identifiers in human and the model system fruit fly. In addition, translation procedures are available to map between genes, transcripts and UniProt identifiers, and pairwise orthology [26] can be inferred between human and the model organisms fruit fly, mouse, and *C. elegans*. The conversion schemes have been implemented generically to facilitate extension to any desired organism.

We have developed a means to establish a link between SNP data (in the form of genomic coordinates) and genes through LD-based haplotype blocks [27]. This is especially useful when analyzing tag SNPs reported in human genome-wide association (GWA) studies, which are considered as a marker for the genes contained in their respective LD block. In addition, genes can be queried for known transcripts and their intron/exon composition.

### Annotation retrieval

Modules in this tool group allow users to annotate genes and proteins with high-level information concerning their functional role in an organism. We report protein-protein interactions or protein complexes based on the iRefIndex database [28], pathway information is made accessible through the Reactome database [29, 30]. In addition, Gene Ontology (GO) [31] annotations are accessed by either querying the official GO database or a local database instance. Finally, tissue specific gene expression data is imported from the GeneAtlas database [32].

One of the ultimate goals of human genomics and proteomics research is to identify relationships between genetic variation and phenotypes. Both ClinVar [33] and the Human Gene Mutation Database (HGMD) [34] provide annotations for human genetic variation and their role in health and disease. These resources are accessible with Dintor. A license is required to access HGMD, and license holders may use the command line version in combination with a local database installation. Drugs and their targets have also been integrated into the Dintor suite by providing access to DrugBank [35]. In addition, pharmacogenomics annotations are available from PharmaADME (http://pharmaadme.org).

### Computational methods

The tools described so far retrieve information from available databases to establish links with data of interest. We have implemented a set of tools that goes beyond data integration, where functional and semantic similarity between pairs of proteins is calculated by applying selected approaches [36]. Furthermore, the Dintor framework provides a means for elementary gene set enrichment analysis [37] based on GO terms. In addition, we have created a gene prioritization module that combines different types of evidence based on GO term annotations, protein-protein interactions and co-complexes, Reactome pathway models, and protein functional similarity through a rank-based approach similar to that implemented in the MetaRanker method [38].

## Results and discussion

In the following four sections we present use-cases demonstrating the capabilities of the Dintor framework to build pipelines and to take advantage of versioned datasets. The use-cases were chosen from recent, representative publications to cover a wide range of Dintor’s functionality. In the first example we illustrate the steps to convert signals from human GWA studies to testable candidate genes from model organisms. We present a simple pipeline that takes dbSNP entries associated with Parkinson’s disease and walk through the steps to find fly orthologs for the genes contained in the respective LD-based haplotype blocks. In the second example, we investigate Dintor’s annotation possibilities by analyzing a small dataset consisting of non-synonymous point mutations associated with autism spectrum disorders [39]. Next, we explore the possibility to use versioned data by repeating a gene set enrichment analysis employing data from 2012. Finally, we compare our gene prioritization tool with commonly used tools based on a benchmark dataset from 2010 [40]. Unix shell script pipelines, input files and result files are included as Additional file 2 for the first three examples.

### Parkinson’s disease GWAS example

Parkinson’s disease (PD) is a neurodegenerative disorder caused by the death of dopamine generating cells in the brain’s substantia nigra, resulting in tremor, bradykinesia, and rigidity. Genes associated with PD can be tested in fruit flies by studying movement in fly lines with silenced orthologous candidate genes. Recently, Nalls et al. [41] published a highly powered GWA study, which lists 26 loci with their respective tag SNPs significantly associated with PD. In this example, we demonstrate the capabilities of the Dintor framework by outlining the steps to obtain orthologous candidate genes in fruit fly for the genes located at these 26 human loci. This pipeline is also available as a tutorial on our Galaxy web service (http://dintor.eurac.edu).

In a first step we take the dbSNP entries from Table 1 of the abovementioned publication and convert these dbSNP identifiers to coordinates on the human genome (version GRCh37). With these coordinates we look up the closest LD-based haplotype block [27] and find that all SNP locations are contained within LD blocks. In the next step we select the genes located within those LD blocks, as these genes might include PD causal variants. The HGNC symbols of those genes are obtained based on their Ensembl identifiers, which are mapped to fly orthologs using Ensembl Compara orthology tables [26]. Two additional conversions based on the orthologs’ FlyBase gene identifiers provide us with the fly annotation symbols (CG numbers) and the respective Vienna Drosophila Resource Center (VDRC) transformant identifiers, which can readily be used to order fly lines for RNAi knock-down experiments.

**Fig. 2:**
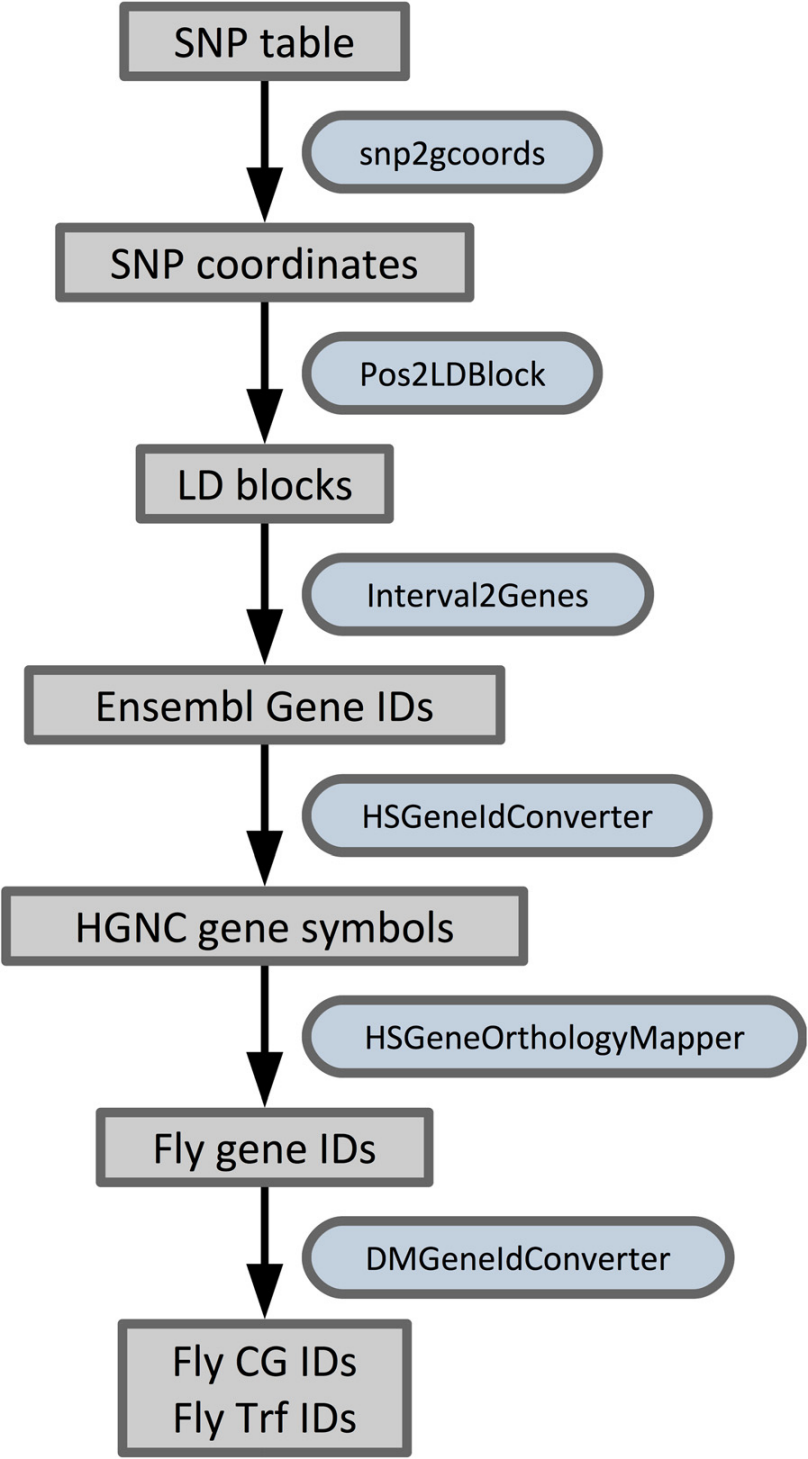
Parkinson’s disease GWA annotation pipeline. Shown here is the workflow for processing the PD GWA input table containing dbSNP identifiers. Gray boxes indicate tabular text files. Boxes with rounded corners and blue background designate file-processing tools accepting as input a table and extending it with additional information by appending new data columns. These tool boxes are labeled with their respective Dintor tool names. Arrows indicate the workflow direction by connecting input and output data files, with the processing tool placed next to the arrow. The pipeline starts with converting dbSNP identifiers from the original table to coordinates referring to the GRCh37 genome, and ends with a double invocation of Dintor’s fly gene identifier converter, DMGeneIdConverter, in order to retrieve fly annotation symbols (CG IDs) and VDRC transformant identifiers (Trf IDs)

In this small example, we started with a table consisting of 26 dbSNP entries, corresponding to the 26 loci identified by the GWA study. Each step was carried out with a distinct tool and added specific information to the original input table (Fig. 2). For cases where multiple hits were reported for a single entity (for example several genes in an LD block, or multiple fly orthologs for a single human gene), each hit was reported in a separate line. To summarize, for the 26 loci/LD blocks we found 79 human Ensembl gene IDs related to 44 fly orthologs, which can be targeted by 106 VDRC fly lines.

### Variant annotation

Many of the Dintor tools are useful for variant annotation as demonstrated in the following example, where we retrieve relevant information for a small set of selected variants. The example is based on a publication reporting the exome sequencing of family trios with idiopathic forms of autism spectrum disorders (ASDs) [39], a mental health disorder characterized by deficits in social communication and interaction, which usually is diagnosed during the first two years of childhood. The study aimed to identify *de novo* private, protein-altering, spontaneous mutations in persons suffering from ASD by sequencing the exomes of the affected individuals and their parents. This study is a representative example where individual level data is used to query various annotation databases, which in many countries is strictly regulated by privacy law. Therefore, utilizing Dintor tools in combination with local database installations provides a means to comply with such legal regulations, as no data are sent to public servers.

**Fig. 3:**
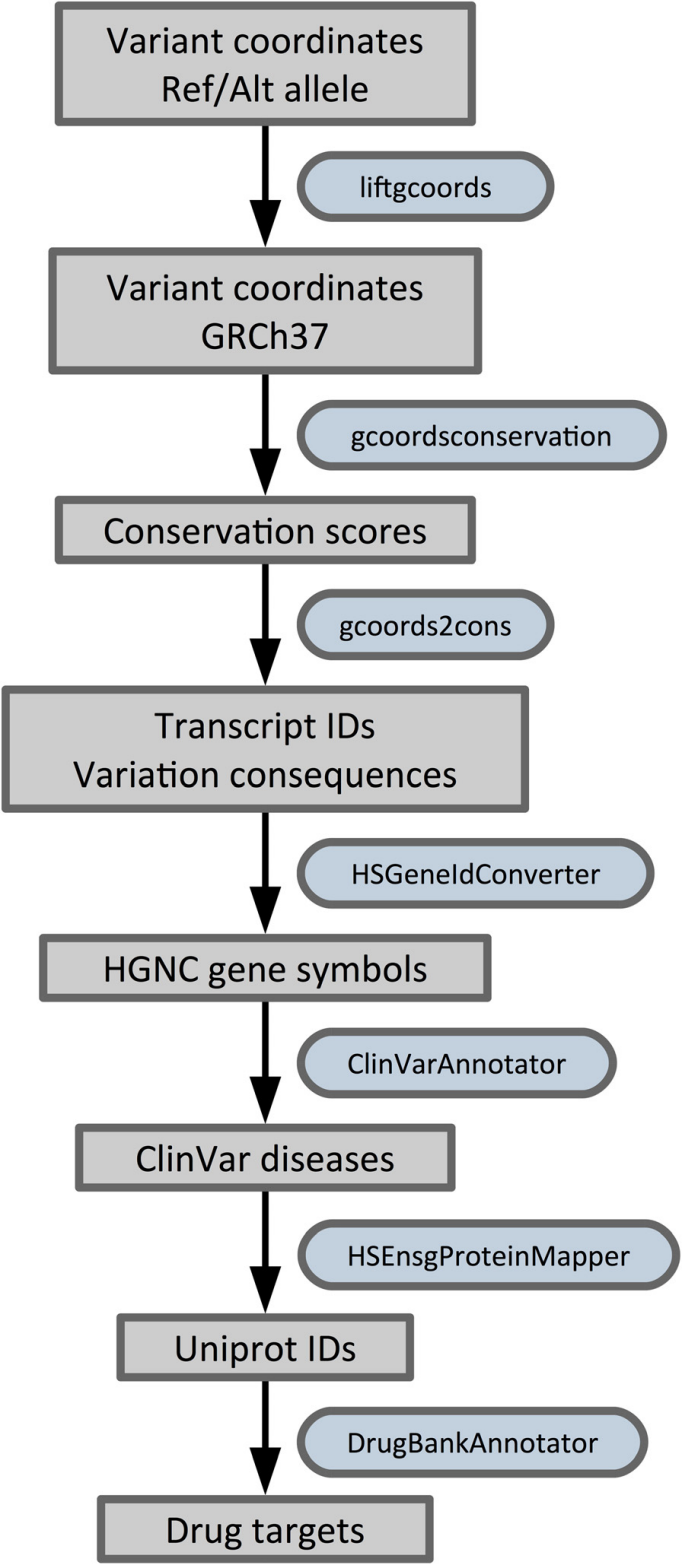
Variant annotation pipeline. This figure illustrates the pipeline for processing three genetic variations identified by exome sequencing as potential causative *de novo* point mutations in sporadic autism spectrum disorders. The symbols used in this figure are the same as in Fig. 2. The analysis starts by lifting the genomic coordinates of the three point mutations from the originally provided NCBI36 coordinates to GRCh37. Conservation and variation consequence information is added before the affected genes are identified. Ultimately, pharmacological information is retrieved for the three proteins affected by each of the point mutations

In the example pipeline (see Fig. 3) we focus on annotating three disruptive *de novo* point mutations that were found to be potentially causative for sporadic ASD in three different genes, as listed in Table 2 of the publication [39]: *LAMC3* (encodes for laminin gamma 3 chain), *SCN1A* (encodes for a voltage gated Na^+^ channel protein), and *GRIN2B* (encodes for an ionotropic glutamate receptor). As many publications from around the year 2011, this ASD study still reports genomic locations based on the NCBI36 genome version. We therefore start with lifting the coordinates to Dintor’s current reference genome, GRCh37. In a following step we retrieve the GERP conservation scores [42] for 37 eutherian mammals from the Ensembl database. For all three mutations we observe conservation scores close to 5.0, an indication that the positions are under evolutionary constraint. We continue the annotation process by investigating the effects of the three mutations with a Dintor tool that reports multiple types of transcript-related information, such as Ensembl transcript identifier, consequence type, codon change, SIFT [22] and PolyPhen2 [23] scores. In all transcripts, the mutations are characterized as highly deleterious by SIFT and PolyPhen2. The mutation in gene *GRIN2B* occurs at a splice site, whereas the other two are missense mutations in exons 5 and 26 of *LAMC3* and *SCN1A*, respectively. There is a single transcript of *LAMC3*, and the respective mutation results in a change of a negatively charged aspartic acid to a glycine with neutral charge. In each of the four protein-coding transcripts of *SCN1A* the respective variation results in a substitution of a proline residue to leucine.

Querying the current ClinVar [33] database with the respective Dintor tool reveals that the splice site mutation in *GRIN2B* is associated with mental retardation. Looking at this annotation historically, it turns out that the ClinVar entry originates from a later publication on ASD by the same group [43].

We ultimately want to find out if the proteins encoded by these three genes appear in a pharmacological context. This can readily be answered by searching the corresponding UniProt accession numbers in the Drugbank [35] database utilizing the respective Dintor tool. No drugs are found to act on LAMC3, but the voltage gated Na^+^ channel protein SCN1A is inhibited mainly by anticonvulsant drugs to treat epilepsy. Protein GRIN2B is targeted mainly by antagonistic drugs, including anticonvulsants for treatment of epilepsy, but also as severe pain reliever or in treatment of schizophrenia and other psychoses. A literature research shows that anticonvulsants prescribed for epilepsy treatment such as topiramate (which acts on SCN1A) have also been investigated as medications for ASDs, however with only small success [44].

### Gene set enrichment analysis

Gene ontology-based gene set enrichment (GSE) is a method to detect over- or underrepresentation of GO terms in a defined set of input genes. We have implemented a simple GSE method applying a hypergeometric statistical model and Fisher’s exact test to detect GO term enrichment, depletion, or both [45]. With this GSE tool, we investigate to which degree of detail we can reproduce a typical enrichment study published in 2012 [46], in which a set of 71 genes was identified to be significantly differentially expressed upon treatment of acute lymphoblastic leukemia (ALL) cell lines with dexamethasone, a synthetic glucocorticoid used during ALL treatment.

We take the HGNC gene symbols from Table 1 in [46] and convert them to Ensembl gene identifiers using the gene identifier conversion tool based on data retrieved from Ensembl release 65, corresponding to the earliest release of our Dintor framework in April 2012. This allows us to rescue three gene symbols (listed as C18orf1, C6orf81, and C7orf40 in the publication, now assigned to gene symbols LDLRAD4, ARMC12, SNHG15), losing C1orf107, which has been renamed to gene symbol DIEXF before Ensembl release 65. A second lost gene is traced back to a misspelling in the article (DPED1). Six additional gene symbols cannot be mapped. These correspond to retired Ensembl gene models (2), non-protein coding genes (3), and one uncharacterized gene. The resulting 63 gene identifiers are then mapped to UniProt accession numbers, which form the input for the GSE tool.

Enrichment is performed with a microarray-specific set of background genes, retrieved from the series matrix of the original article, deposited with Gene Expression Omnibus identifier GSE29003. In order to replicate the results we search for enriched GO terms in the biological process ontology, reporting Benjamini-Hochberg [47] corrected *p*-values at a false discovery rate of 5 %, they are listed in Table 1.

**Table 1:** Results from acute lymphoblastic leukemia gene set enrichment analysis based on GO biological process ontology

GO term^a^	*p*-value^c^	GO term name
**GO:0042981** ^b^	2.01 × 10^−5^	Regulation of apoptotic process
GO:0006915^b^	6.29 × 10^−5^	Apoptotic process
**GO:0010942**	1.95 × 10^−4^	Positive regulation of cell death
GO:0008219^b^	3.57 × 10^−4^	Cell death
**GO:0043065**	5.23 × 10^−4^	Positive regulation of apoptotic process
GO:0043402	2.49 × 10^−3^	Glucocorticoid mediated signaling pathway
GO:1902532	4.78 × 10^−3^	Negative regulation of intracellular signal transduction
**GO:0010033**	1.42 × 10^−2^	Response to organic substance
GO:2000271	2.61 × 10^−2^	Positive regulation of fibroblast apoptotic process
GO:0007517	2.82 × 10^−2^	Muscle organ development
GO:0090073^b^	2.82 × 10^−2^	Positive regulation of protein homodimerization activity
**GO:0009968**	3.13 × 10^−2^	Negative regulation of signal transduction
GO:0007519	3.29 × 10^−2^	Skeletal muscle tissue development
GO:0014902	3.33 × 10^−2^	Myotube differentiation
GO:0009966	4.24 × 10^−2^	Regulation of signal transduction
GO:0043523	4.24 × 10^−2^	Regulation of neuron apoptotic process
GO:0045663	4.24 × 10^−2^	Positive regulation of myoblast differentiation
GO:0048011	4.24 × 10^−2^	Neurotrophin TRK receptor signaling pathway
GO:0048741	4.24 × 10^−2^	Skeletal muscle fiber development
GO:0002260	4.50 × 10^−2^	Lymphocyte homeostasis
GO:0046426	4.50 × 10^−2^	Negative regulation of JAK-STAT cascade
GO:1901216	4.50 × 10^−2^	Positive regulation of neuron death
GO:0021542	4.83 × 10^−2^	Dentate gyrus development
GO:0014070	4.88 × 10^−2^	Response to organic cyclic compound

^a^GO terms emphasized in bold letters refer to terms that have been listed in Table 2 of [46]

^b^Enriched terms found by our GSE tool when carrying out the analysis with GO data from January 2012 (date of publication)

^c^Listed *p*-values are Benjamini-Hochberg adjusted and restricted to values lower than 0.05

The results indicate that the enrichment tool clearly identifies the apoptosis process as discussed in the original work. Running the analysis with recent GO data (July 2014) yields a larger number of overrepresented GO terms compared to performing the analysis with data from early 2012. In both cases however, the analysis identifies highly significantly enriched GO terms equal to those ranked high in the original work. Our results are also in agreement with enrichment analysis output obtained from the current Gene Ontology web site, http://amigo.geneontology.org/rte (data not shown).

### Gene prioritization

The goal of gene prioritization is to identify the most relevant genes for a particular phenotype or a disease of interest, from a large set of input candidates, for example, resulting from a high-throughput genomics experiment. A multitude of computational prioritization methods have been developed in recent years using different data types and integration methods; eight popular tools have been evaluated by Börnigen et al. in 2010 [40]. To establish a common benchmark dataset, the authors first reviewed the literature for previously unknown disease-gene associations and defined a validation dataset with 42 disease genes. For each of the test cases, they identified genes already associated with the respective disease (training genes), collected disease-related keywords from databases such as OMIM [48], and gathered input candidates by taking all genes from a 10Mbp region around each disease gene in the validation set. Then they queried each prioritization tool and computed different rank-based performance measures Table 9 in [40]).

In order to correctly assess the performance of the Dintor prioritization tool, it is crucial to use the same data that were available in May 2010, when Börnigen et al. performed their literature review. If newer data were used instead, we might benefit from a ”knowledge contamination” [49], where the publication that reported the disease association has later been incorporated into databases such as GO or Reactome, which are then used in the prioritization. This would result in overly optimistic results and does not reproduce a real application scenario.

Dintor’s prioritization tool can be instructed to use different types of evidence; we used the default setting that incorporates protein-protein interactions and co-complex associations (obtained from iRefIndex v7.0), functional annotations and functional similarity (using GO release 2010.05), and biochemical reactions and pathways (Reactome release 32) into an overall ranking.

The newer Ensembl release 75 was used for creating the training and candidate gene sets by mapping the gene symbols provided in Supplementary Table 3 of Börnigen et al. [40], and for retrieving the candidate genes from the 10Mbp genomic regions around the disease genes, provided in the same table. Spurious mappings were manually resolved.

We assessed our tool by computing the same performance measures used by Börnigen et al., in particular, response rate, median of all rank ratios, and in how many of the 42 test cases the disease gene was reported in the top 5 % (true positive rate (TPR) in top 5 %), top 10 %, and top 30 % of all ranked candidates (see Table 2). The choice of the right measure to judge the performance of a method depends on the particular task. Given that limited resources commonly restrict the number of candidates that can be subjected to further experimental validation, we argue that the measures that indicate top positions (TPR in top 5 % and 10 %) are of more practical relevance than those indicating the overall distribution (TPR in top 30 % and median). Dintor achieves good results when evaluated using measures of the former category, in TPR in top 5 % only ToppGene [16] achieves a better performance. Dintor relies to a considerable extent on GO annotations for prioritization, and the sparse annotations in the older 2010 annotation data might explain the poor median performance. It would be of great interest to further investigate the relative strengths and weaknesses of the different prioritization tools based on an updated benchmark test set.

**Table 2:** Performance of Dintor gene prioritization tool compared to results reported in [40]

Tool name^a^	Response rate	TPR in top 5 %	TPR in top 10 %	TPR in top 30 %	Median
Candid [51]	100 %	21.4 %	33.3 %	64.3 %	18.11
Dintor	100 %	31.0 %	42.9 %	59.5 %	23.62
Endeavour-CS [52]	100 %	26.2 %	42.9 %	90.5 %	11.16
Endeavour-GW [52]	100 %	28.6 %	38.1 %	71.4 %	15.49
GeneDistiller [53]	97.6 %	26.2 %	47.6 %	78.6 %	11.11
GeneWanderer-DK [54]	88.1 %	11.9 %	21.4 %	52.4 %	22.97
GeneWanderer-RW [54]	95.2 %	16.7 %	26.2 %	61.9 %	22.11
Pinta-CS [55]	100 %	28.6 %	31.0 %	71.4 %	18.87
Pinta-GW [55]	100 %	26.2 %	31.0 %	71.4 %	19.03
ToppGene [16]	97.6 %	35.7 %	42.9 %	52.4 %	16.80

Tools that were reported to have a response rate lower than 80 % were not included. The following abbreviations are used: *CS* candidate set, *GW* genome-wide, *TPR* true positive rate

^a^Tool names were taken from [40], references associated with tools are provided in square brackets next to their names. The table is sorted alphabetically by tool name

This example shows that Dintor’s gene prioritization tool achieves a performance comparable to state-of-the-art prioritization suites when the goal is to select top ranking candidates, and it also demonstrates the capacity to perform analyses using datasets from a user specified point in time.

A simple gene prioritization example is provided in the tutorial section of our Galaxy web service (http://dintor.eurac.edu).

## Conclusions

We describe Dintor, a data integration framework for the analysis of genomics and proteomics data. Dintor provides multiple tools for characterizing and annotating genes and their products, as well as methods for investigating gene/protein relationships and candidate prioritization. The underlying datasets used in the analysis can be exchanged by design, allowing more control by the user and better reproducibility of results. Multiple interfaces are provided to fulfill the needs of users with different demands in terms of usability, functionality, and control. Dintor is available as a public web service but can also be installed locally for improved performance and data security under an open-source license.

Dintor will be updated regularly and developed further. In particular, we intend to include additional databases covering pathways and molecular interactions, and we plan to include additional ontologies, like the Mammalian Phenotype Ontology [50]. We also intend to further develop methods for investigating relationships between candidate genes/proteins. Finally, we encourage future collaborative software development based on the open source licensing model.

## Availability and requirements

Project name: Dintor

Project home page: http://dintor.eurac.edu (free source code and free web service)

Operating systems: Linux and Mac OS X

Programming languages: Python and Perl

Other requirements: Galaxy [5] and Apache, when running as a local web server

License: The software is published under the MIT License

Any restrictions to use by non-academics: None

Ethics approval: No ethics approval was required for this work.

## Additional files

Additional file 1:
**Dintor tools summary.** (PDF 567 kb) Additional file 2:
**Data files for Dintor use-cases one to three.**

## Abbreviations

ALL: Acute lymphoblastic leukemia
ASD: Autism spectrum disorders
CS: Candidate set
GO: Gene Ontology
GSE: Gene set enrichment
GWA: Genome-wide association
HGNC: Human genome organisation gene nomenclature committee
HGMD: Human gene mutation database
LD: Linkage disequilibrium
PD: Parkinson’s disease
SNP: Single nucleotide polymorphism
TPR: True positive rate
VDRC: Vienna drosophila resource center

## References

[1] Kumar S, Dudley J (2007). Bioinformatics software for biologists in the genomics era. Bioinformatics.

[2] Gentleman R (2005). Reproducible research: a bioinformatics case study. Stat Appl Genet Mol Biol.

[3] Mesirov JP (2010). Computer science. Accessible reproducible research. Science.

[4] Wolstencroft K, Haines R, Fellows D, Williams A, Withers D, Owen S (2013). The Taverna workflow suite: designing and executing workflows of Web Services on the desktop, web or in the cloud. Nucleic Acids Res.

[5] Goecks J, Nekrutenko A, Taylor J, Galaxy Team (2010). Galaxy: a comprehensive approach for supporting accessible, reproducible, and transparent computational research in the life sciences. Genome Biol.

[6] Reich M, Liefeld T, Gould J, Lerner J, Tamayo P, Mesirov JP (2006). GenePattern 2.0. Nat Genet.

[7] Lushbough CM, Jennewein DM, Brendel VP (2011). The BioExtract server: a web-based bioinformatic workflow platform. Nucleic Acids Res.

[8] Deelman E, Gannon D, Shields M, Taylor I (2009). Workflows and e-science: an overview of workflow system features and capabilities. Future Gener Comp Sy.

[9] Curcin V, Ghanem M (2008). Scientific workflow systems - can one size fit all? in: Biomedical Engineering Conference. Cairo: IEEE.

[10] Gentleman RC, Carey VJ, Bates DM, Bolstad B, Dettling M, Dudoit S (2004). Bioconductor: open software development for computational biology and bioinformatics. Genome Biol.

[11] Azam S, Rathore A, Shah TM, Telluri M, Amindala B, Ruperao P (2014). An integrated SNP mining and utilization (ISMU) pipeline for next generation sequencing data. PLoS One.

[12] Nagasaki M, Fujita A, Sekiya Y, Saito A, Ikeda E, Li C (2013). XiP: a computational environment to create, extend and share workflows. Bioinformatics.

[13] Muñiz-Fernandez F, Carreño-Torres A, Morcillo-Suarez C, Navarro A (2011). Genome-wide association studies pipeline (GWASpi): a desktop application for genome-wide SNP analysis and management. Bioinformatics.

[14] Huang DW, Sherman BT, Lempicki RA (2009). Systematic and integrative analysis of large gene lists using DAVID bioinformatics resources. Nat Protoc.

[15] Kamburov A, Stelzl U, Lehrach H, Herwig R (2013). The ConsensusPathDB interaction database: 2013 update. Nucleic Acids Res.

[16] Chen J, Xu H, Aronow BJ, Jegga AG (2007). Improved human disease candidate gene prioritization using mouse phenotype. BMC Bioinf.

[17] Triplet T, Butler G (2014). A review of genomic data warehousing systems. Brief Bioinform.

[18] Smedley D, Haider S, Ballester B, Holland R, London D, Thorisson G (2009). BioMart-biological queries made easy. BMC Genomics.

[19] Cunningham F, Amode MR, Barrell D, Beal K, Billis K, Brent S (2015). Ensembl 2015. Nucleic Acids Res.

[20] Sherry ST, Ward MH, Kholodov M, Baker J, Phan L, Smigielski EM (2001). dbSNP: the NCBI database of genetic variation. Nucleic Acids Res.

[21] Lewontin RC (1988). On measures of gametic disequilibrium. Genetics.

[22] Ng PC, Henikoff S (2003). SIFT: Predicting amino acid changes that affect protein function. Nucleic Acids Res.

[23] Adzhubei IA, Schmidt S, Peshkin L, Ramensky VE, Gerasimova A, Bork P (2010). A method and server for predicting damaging missense mutations. Nat Methods.

[24] González-Pérez A, López-Bigas N (2011). Improving the assessment of the outcome of nonsynonymous SNVs with a consensus deleteriousness score, Condel. Am J Hum Genet.

[25] Kellis M, Wold B, Snyder MP, Bernstein BE, Kundaje A, Marinov GK (2014). Defining functional DNA elements in the human genome. Proc Natl Acad Sci U S A.

[26] Vilella AJ, Severin J, Ureta-Vidal A, Heng L, Durbin R, Birney E (2009). EnsemblCompara GeneTrees: complete, duplication-aware phylogenetic trees in vertebrates. Genome Res.

[27] Taliun D, Gamper J, Pattaro C (2014). Efficient haplotype block recognition of very long and dense genetic sequences. BMC Bioinf.

[28] Razick S, Magklaras G, Donaldson IM (2008). iRefIndex: a consolidated protein interaction database with provenance. BMC Bioinf.

[29] Croft D, Mundo AF, Haw R, Milacic M, Weiser J, Wu G (2014). The reactome pathway knowledgebase. Nucleic Acids Res.

[30] Milacic M, Haw R, Rothfels K, Wu G, Croft D, Hermjakob H (2012). Annotating cancer variants and anti-cancer therapeutics in reactome. Cancers (Basel).

[31] Ashburner M, Ball CA, Blake JA, Botstein D, Butler H, Cherry JM (2000). Gene ontology: tool for the unification of biology. The gene ontology consortium. Nat Genet.

[32] Su AI, Wiltshire T, Batalov S, Lapp H, Ching KA, Block D (2004). A gene atlas of the mouse and human protein-encoding transcriptomes. Proc Natl Acad Sci U S A.

[33] Landrum MJ, Lee JM, Riley GR, Jang W, Rubinstein WS, Church DM (2014). ClinVar: public archive of relationships among sequence variation and human phenotype. Nucleic Acids Res.

[34] Stenson PD, Ball EV, Mort M, Phillips AD, Shiel JA, Thomas NST (2003). Human gene mutation database (HGMD): 2003 update. Hum Mutat.

[35] Wishart DS, Knox C, Guo AC, Cheng D, Shrivastava S, Tzur D (2008). DrugBank: a knowledgebase for drugs, drug actions and drug targets. Nucleic Acids Res.

[36] Guzzi PH, Mina M, Guerra C, Cannataro M (2012). Semantic similarity analysis of protein data: assessment with biological features and issues. Brief Bioinform.

[37] Khatri P, Draghici S (2005). Ontological analysis of gene expression data: current tools, limitations, and open problems. Bioinformatics.

[38] Pers TH, Hansen NT, Lage K, Koefoed P, Dworzynski P, Miller ML (2011). Meta-analysis of heterogeneous data sources for genome-scale identification of risk genes in complex phenotypes. Genet Epidemiol.

[39] O’Roak BJ, Deriziotis P, Lee C, Vives L, Schwartz JJ, Girirajan S (2011). Exome sequencing in sporadic autism spectrum disorders identifies severe de novo mutations. Nat Genet.

[40] Börnigen D, Tranchevent L-C, Bonachela-Capdevila F, Devriendt K, De Moor B, De Causmaecker P (2012). An unbiased evaluation of gene prioritization tools. Bioinformatics.

[41] Nalls MA, Pankratz N, Lill CM, Do CB, Hernandez DG, Saad M (2014). Large-scale meta-analysis of genome-wide association data identifies six new risk loci for Parkinson’s disease. Nat Genet.

[42] Cooper GM, Stone EA, Asimenos G, Green ED, Batzoglou S, NISC Comparative Sequencing Program (2005). Distribution and intensity of constraint in mammalian genomic sequence. Genome Res.

[43] O’Roak BJ, Vives L, Fu W, Egertson JD, Stanaway IB, Phelps IG (2012). Multiplex targeted sequencing identifies recurrently mutated genes in autism spectrum disorders. Science.

[44] Hirota T, Veenstra-Vanderweele J, Hollander E, Kishi T (2014). Antiepileptic medications in autism spectrum disorder: a systematic review and meta-analysis. J Autism Dev Disord.

[45] Rivals I, Personnaz L, Taing L, Potier MC (2007). Enrichment or depletion of a GO category within a class of genes: which test?. Bioinformatics.

[46] Rainer J, Lelong J, Bindreither D, Mantinger C, Ploner C, Geley S (2012). Research resource: transcriptional response to glucocorticoids in childhood acute lymphoblastic leukemia. Mol Endocrinol.

[47] Benjamini Y, Hochberg Y (1995). Controlling the false discovery rate: a practical and powerful approach to multiple testing. J R Stat Soc Ser B Methodol.

[48] Amberger JS, Bocchini CA, Schiettecatte F, Scott AF, Hamosh A (2015). OMIM.org: online Mendelian inheritance in Man (OMIM®), an online catalog of human genes and genetic disorders. Nucleic Acids Res.

[49] Moreau Y, Tranchevent LC (2012). Computational tools for prioritizing candidate genes: boosting disease gene discovery. Nat Rev Genet.

[50] Smith CL, Goldsmith CA, Eppig JT (2005). The mammalian phenotype ontology as a tool for annotating, analyzing and comparing phenotypic information. Genome Biol.

[51] Hutz JE, Kraja AT, McLeod HL, Province MA (2008). CANDID: a flexible method for prioritizing candidate genes for complex human traits. Genet Epidemiol.

[52] Aerts S, Lambrechts D, Maity S, Van Loo P, Coessens B, De Smet F (2006). Gene prioritization through genomic data fusion. Nat Biotechnol.

[53] Seelow D, Schwarz JM, Schuelke M (2008). GeneDistiller-distilling candidate genes from linkage intervals. PLoS One.

[54] Köhler S, Bauer S, Horn D, Robinson PN (2008). Walking the interactome for prioritization of candidate disease genes. Am J Hum Genet.

[55] Nitsch D, Gonçalves JP, Ojeda F, de Moor B, Moreau Y (2010). Candidate gene prioritization by network analysis of differential expression using machine learning approaches. BMC Bioinf.

