# Supplementary material for: Dintor: functional annotation of genomic and proteomic data
Source: BMC Genomics. 2015 Dec 21;16:1081. doi: 10.1186/s12864-015-2279-5 (PMC4687148; doi:10.1186/s12864-015-2279-5)
Supplement: Additional file 1: — Dintor tools summary. (PDF 567 kb) [file 12864_2015_2279_MOESM1_ESM.pdf]

# Dintor: Functional Annotation of Genomic and Proteomic Data (Supplementary Data)

Christian X. Weichenberger, Hagen Blankenburg, Antonia Palermo, Yuri D'Elia, Eva König, Erik Bernstein and Francisco S. Domingues

This document provides a comprehensive list of Dintor tools, along with a short description of each tool and tool-specific references to scientific literature explaining the methodology utilized in the respective tool. The list is split into the three categories used by the Dintor Galaxy web server, and the order of tool appearance is identical to that on the web server as of December 2015, facilitating individual tool lookup. The functionality provided in the Galaxy interface is a simpler version of the capabilities offered by the wrapped command line tools. We therefore also refer to the "Full reference" help page URL provided at the end of a Galaxy Dintor tool or the `--help` option when running the tool from the command line.

## Basic functionality

| Dintor tool name | Description | References |
|---|---|---|
| *DMGeneIdConverter* | Convert between *Drosophila* gene identifiers: FlyBase gene ID (FBgn*), fly annotation symbols (CG*), and Vienna Drosophila Resource Center (VDRC) transformant ID. Various VDRC libraries are supported. | [1], [2] |
| *gcoords2cons* | Retrieve consequence types for *Homo sapiens* mutations from pairs of reference/alternate alleles for genomic coordinates. Outputs Ensembl consequence type, transcript ID, SIFT, PolyPhen and Condel scores, and HGVS nomenclature for protein coding mutations. | [3], [4], [5] |
| *Gcoords2gcoords* | Convenience script to convert between Dintor genomic coordinates (GC) and other commonly used GC formats. | |
| *gcoords2genes* | Query for human genes in the vicinity of a position on the genome, usually addressing a variation. Output includes the Ensembl human gene ID and the distance to the gene, its strand and a distance-based rank. | |
| *gcoords2ld* | Compute linkage disequilibrium (LD) between pairs of GCs or a GC and a gene. Outputs *D'* and $r^2$ measures. For a GC/gene pair, all SNPs from the gene are taken and the maximum LD measure is reported. Calculation can be restricted to certain populations available in the 1000 Genomes/HapMap projects. | [6] |
| *gcoords2reg* | Query for regulatory regions from the Encode project for a position on the genome. The level of output detail can be chosen and reflects the way Encode data are organized. | [7] |
| *gcoords2snp* | Inverse function of *snp2gcoords*. Queries a position on the genome for dbSNP entries and outputs any rs* IDs found. | [8] |
| *gcoordsconservation* | Query if a given position on the genome is located in a conserved regions across a selectable set of organisms. Depending on the choice of conservation assignment, output is either binary (conserved or not) or a GERP score. | [9], [10] |
| *gene2canonexons* | Retrieve a list of canonical exons for a human gene. Useful for producing input for Illumina DesignStudio software. | |
| *liftgcoords* | Lift genomic coordinates from a previous human genome release to the current. | |

| | | |
|---|---|---|
| *snp2gcoords* | Convert dbSNP rs* IDs to genomic coordinates as used in Dintor. Output includes Ensembl quality control flags, reference and alternate alleles, strand information, and evidence backing up the SNP. Inverse functionality is given by *gcoords2snp*. | [8] |
| *tbl2tbl* | Import filter for tabular data. Used to import arbitrarily formatted text-based tables into Dintor's tab-separated format. | |
| *tblsubmerge* | Merge a table based on a column's unique identifier. Rows with the same entry in a specified column will be joined into a single row. | |
| *tblsubsplit* | Split single or multiple cells into separate rows, optionally adding an index column that can be used to undo this operation by calling *tblsubmerge*. | |
| *HSEnsgProteinMapper* | Human gene ↔ protein mapping tool. Maps between any combination of Ensembl gene ID, consensus coding sequence (CCDS), Ensembl transcript ID, Ensembl protein ID, UniProt SwissProt or Trembl accession number or entry name. A common use case is mapping from Ensembl gene IDs (or transcript IDs) derived from Dintor tools to UniProt or CCDS. | [11], [12] |
| *HSGeneIdConverter* | Converts between widely used gene identifiers in human, including Ensembl gene IDs, NCBI Entrez IDs, HUGO Gene Nomenclature Committee (HGNC) gene symbols. | [13], [14] |
| *HSGeneOrthologyMapper* | Derive orthology information between human genes and the most common scientific model organisms, fruit fly, mouse, and worm. Orthology mappings are based on Ensembl. | [15] |
| *Interval2Genes* | List human genes contained in a genomic region specified by a pair of begin/end GCs. Additionally, it can be used to relate a genomic position (as originating from a variation) to the genes contained in an LD block output by the *Pos2LDBlock* tool. | |
| *Pos2LDBlock* | Assign LD-based haplotype blocks to a position on the genome. The output encodes the relationship between the query position and the LD-haplotype block. | [16] |
| *TableJoiner* | Join two tables on a common column. Unlike the Unix *join* command, this tool works on arbitrary, unsorted, tab-separated tables and allows transferring subsets of columns from the joined table. | |
| *VCF2Dint* | Import filter for variant call format (VCF) files into Dintor tables. | |

## Annotation retrieval

| Dintor tool name | Description | References |
|---|---|---|
| *ClinVarAnnotator* | Output hits in NCBI ClinVar database for a GC, a GC with reference and alternate allele, or an interval on a chromosome (e.g. derived from *Interval2Genes*). | [17] |
| *DrugBankAnnotator* | Retrieve DrugBank information for UniProt accession numbers. Lists drugs associated with proteins identified by their respective UniProt accession numbers. | [18] |
| *GOAnnotator* | Query Gene Ontology (GO) for either GO terms and their descendants or for GO terms associated with UniProt accession numbers. A variety of information can be retrieved, such as GO term names, evidence codes, and ontology name. Filters exist to limit the number of terms to a certain depth in the GO graph, to ontologies, to certain types of edges, and to high quality SwissProt entries. | [19] |
| *HGMDAnnotator* | [**not available on public Galaxy web server**] The human gene mutation database (HGMD) contains manual annotations of human gene mutations. Due to licensing restrictions, access to this database is only available as a command line tool interfacing a purchased HGMD MySQL database. The tool itself has an interface comparable to *ClinVarAnnotator*, and a Galaxy interface is ready for license holders running their own Galaxy server. | [20] |
| *HSGeneAtlas* | Retrieve tissue-specific gene expression for human genes using the Genomics Institute of the Novartis Research Foundation (GNF) Gene Atlas. Filters are available for gene over-, and/or under-expression and tissue types. | [21] |
| *InteractionAnnotator* | Find protein interaction partners using the iRefIndex database. Additional data characterizing the interactions, such as external references and experimental detection techniques, can optionally be output. Interactions may be restricted to a panel of predefined genes/proteins. | [22] |
| *PharmaADMEntor* | Highlight mutations in an industry-initiated database of genetic biomarkers reliably involved in drug metabolism. | [23] |
| *ReactomeAnnotator* | Retrieve information about pathways, reactions, and participating molecules from the Reactome database, taking into account the hierarchical (parent-child) structure of the data. The tool can be queried by UniProt accession numbers, Reactome identifiers, or free text; the output may be restricted to a predefined panel. | [24] |

## Computational methods

| Dintor tool name | Description | References |
|---|---|---|
| *GOEnricher* | Perform GO term-based gene set enrichment analysis. Enrichment can be performed on any of the three ontologies (biological process, molecular function, and cellular component). Correction for multiple hypothesis testing and result clustering are available. Enriched GO terms are usually based on the set originating from all genes, but can also be broken down to genes. | [25], [26] |
| *GOFunSim* | Compute pairwise protein functional similarity. The tool offers calculation of five different functional similarity measures based on six different semantic similarity measures. In addition, functional similarity can be computed between a list of proteins and predefined panel of (usually related) proteins. Furthermore, semantic similarity can be derived for pairs of GO terms. Graph-based GO term information content can also be output. | [27] |
| *MendelianFilter* | Remove variants that do not comply with a certain mode of Mendelian inheritance. The tool operates on a multi-sample VCF file and furthermore requires relatedness be provided in a pedigree (PED format). Filtering is possible for autosomal dominant or recessive, X-linked dominant or recessive, and mitochondrial linked inheritance. | [28], [29] |
| *MetaRanker* | Given an object (e.g. gene) associated with multiple scores, each one in a single column of a table, compute a single, rank-based score from these columns. This module is used in the final ranking provided by *Prioritizer*. Columns may contain missing values, the ordering of a column's content can be specified individually, and the final rank calculation allows weighting the contributing columns. | [30] |
| *Prioritizer* | Performs candidate gene prioritization by a guilt-by-association approach. Candidate genes are compared to a user-defined panel of related genes (e.g. disease associated) by the following Dintor tools:<br>• *InteractionAnnotator*: Does the candidate gene interact with a panel gene?<br>• *ReactomeAnnotator*: Does the candidate gene share pathways with the panel genes?<br>• *GOFunSim*: Is there high functional similarity between the candidate gene and genes from the panel?<br>• *GOAnnotator*: Is the candidate gene involved in similar GO classes as the panel genes? | [31] |

## References


1. dos Santos G, Schroeder AJ, Goodman JL, Strelets VB, Crosby MA, Thurmond J, Emmert DB, Gelbart WM, FlyBase C: **FlyBase: introduction of the Drosophila melanogaster Release 6 reference genome assembly and large-scale migration of genome annotations**. *Nucleic Acids Res* 2015, **43**(Database issue):D690-697.
2. Dietzl G, Chen D, Schnorrer F, Su KC, Barinova Y, Fellner M, Gasser B, Kinsey K, Oppel S, Scheiblauer S *et al*: **A genome-wide transgenic RNAi library for conditional gene inactivation in Drosophila**. *Nature* 2007, **448**(7150):151-156.
3. Ng PC, Henikoff S: **SIFT: Predicting amino acid changes that affect protein function**. *Nucleic Acids Res* 2003, **31**(13):3812-3814.
4. Adzhubei IA, Schmidt S, Peshkin L, Ramensky VE, Gerasimova A, Bork P, Kondrashov AS, Sunyaev SR: **A method and server for predicting damaging missense mutations**. *Nature methods* 2010, **7**(4):248-249.
5. González-Pérez A, López-Bigas N: **Improving the assessment of the outcome of nonsynonymous SNVs with a consensus deleteriousness score, Condel**. *American journal of human genetics* 2011, **88**(4):440-449.
6. Lewontin RC: **On measures of gametic disequilibrium**. *Genetics* 1988, **120**(3):849-852.
7. Kellis M, Wold B, Snyder MP, Bernstein BE, Kundaje A, Marinov GK, Ward LD, Birney E, Crawford GE, Dekker J *et al*: **Defining functional DNA elements in the human genome**. *Proc Natl Acad Sci U S A* 2014, **111**(17):6131-6138.
8. Sherry ST, Ward MH, Kholodov M, Baker J, Phan L, Smigielski EM, Sirotkin K: **dbSNP: the NCBI database of genetic variation**. *Nucleic Acids Res* 2001, **29**(1):308-311.
9. Paten B, Herrero J, Beal K, Fitzgerald S, Birney E: **Enredo and Pecan: genome-wide mammalian consistency-based multiple alignment with paralogs**. *Genome Res* 2008, **18**(11):1814-1828.
10. Cooper GM, Stone EA, Asimenos G, Program NISCCS, Green ED, Batzoglou S, Sidow A: **Distribution and intensity of constraint in mammalian genomic sequence**. *Genome Res* 2005, **15**(7):901-913.
11. Pruitt KD, Harrow J, Harte RA, Wallin C, Diekhans M, Maglott DR, Searle S, Farrell CM, Loveland JE, Ruef BJ *et al*: **The consensus coding sequence (CCDS) project: Identifying a common protein-coding gene set for the human and mouse genomes**. *Genome Res* 2009, **19**(7):1316-1323.
12. UniProt Consortium T: **UniProt: a hub for protein information**. *Nucleic Acids Res* 2015, **43**(Database issue):D204-212.
13. Maglott D, Ostell J, Pruitt KD, Tatusova T: **Entrez Gene: gene-centered information at NCBI**. *Nucleic Acids Res* 2011, **39**(Database issue):D52-57.
14. Gray KA, Yates B, Seal RL, Wright MW, Bruford EA: **Genenames.org: the HGNC resources in 2015**. *Nucleic Acids Res* 2015, **43**(Database issue):D1079-1085.
15. Vilella AJ, Severin J, Ureta-Vidal A, Heng L, Durbin R, Birney E: **EnsemblCompara GeneTrees: Complete, duplication-aware phylogenetic trees in vertebrates**. *Genome Res* 2009, **19**(2):327-335.
16. Taliun D, Gamper J, Pattaro C: **Efficient haplotype block recognition of very long and dense genetic sequences**. *BMC Bioinformatics* 2014, **15**:10.

17. Landrum MJ, Lee JM, Riley GR, Jang W, Rubinstein WS, Church DM, Maglott DR: **ClinVar: public archive of relationships among sequence variation and human phenotype**. *Nucleic Acids Res* 2014, **42**(Database issue):D980-D985.
18. Wishart DS, Knox C, Guo AC, Cheng D, Shrivastava S, Tzur D, Gautam B, Hassanali M: **DrugBank: a knowledgebase for drugs, drug actions and drug targets**. *Nucleic Acids Res* 2008, **36**(Database issue):D901-D906.
19. Ashburner M, Ball CA, Blake JA, Botstein D, Butler H, Cherry JM, Davis AP, Dolinski K, Dwight SS, Eppig JT *et al*: **Gene ontology: tool for the unification of biology. The Gene Ontology Consortium**. *Nat Genet* 2000, **25**(1):25-29.
20. Stenson PD, Ball EV, Mort M, Phillips AD, Shiel JA, Thomas NST, Abeysinghe S, Krawczak M, Cooper DN: **Human Gene Mutation Database (HGMD): 2003 update**. *Hum Mutat* 2003, **21**(6):577-581.
21. Su AI, Wiltshire T, Batalov S, Lapp H, Ching KA, Block D, Zhang J, Soden R, Hayakawa M, Kreiman G *et al*: **A gene atlas of the mouse and human protein-encoding transcriptomes**. *Proc Natl Acad Sci U S A* 2004, **101**(16):6062-6067.
22. Razick S, Magklaras G, Donaldson IM: **iRefIndex: a consolidated protein interaction database with provenance**. *BMC Bioinformatics* 2008, **9**:405.
23. **PharmaADME.org** [http://pharmaadme.org]
24. Croft D, Mundo AF, Haw R, Milacic M, Weiser J, Wu G, Caudy M, Garapati P, Gillespie M, Kamdar MR *et al*: **The Reactome pathway knowledgebase**. *Nucleic Acids Res* 2014, **42**(Database issue):D472-D477.
25. Rivals I, Personnaz L, Taing L, Potier MC: **Enrichment or depletion of a GO category within a class of genes: which test?** *Bioinformatics* 2007, **23**(4):401-407.
26. Khatri P, Draghici S: **Ontological analysis of gene expression data: current tools, limitations, and open problems**. *Bioinformatics* 2005, **21**(18):3587-3595.
27. Guzzi PH, Mina M, Guerra C, Cannataro M: **Semantic similarity analysis of protein data: assessment with biological features and issues**. *Brief Bioinform* 2012, **13**(5):569-585.
28. Sincan M, Simeonov DR, Adams D, Markello TC, Pierson TM, Toro C, Gahl WA, Boerkoel CF: **VAR-MD: a tool to analyze whole exome-genome variants in small human pedigrees with mendelian inheritance**. *Hum Mutat* 2012, **33**(4):593-598.
29. Li MX, Gui HS, Kwan JS, Bao SY, Sham PC: **A comprehensive framework for prioritizing variants in exome sequencing studies of Mendelian diseases**. *Nucleic Acids Res* 2012, **40**(7):e53.
30. Pers TH, Hansen NT, Lage K, Koefoed P, Dworzynski P, Miller ML, Flint TJ, Mellerup E, Dam H, Andreassen OA *et al*: **Meta-analysis of heterogeneous data sources for genome-scale identification of risk genes in complex phenotypes**. *Genet Epidemiol* 2011, **35**(5):318-332.
31. Aerts S, Lambrechts D, Maity S, Van Loo P, Coessens B, De Smet F, Tranchevent L-C, De Moor B, Marynen P, Hassan B *et al*: **Gene prioritization through genomic data fusion**. *Nat Biotechnol* 2006, **24**(5):537-544.
